# The Association Between Autistic Traits and Disordered Eating is Moderated by Sex/Gender and Independent of Anxiety and Depression

**DOI:** 10.1007/s10803-020-04669-z

**Published:** 2020-08-27

**Authors:** Alana Barnett, Katie Edwards, Rebecca Harper, Elizabeth Evans, Derina Alexander, Mohini Choudhary, Gareth Richards

**Affiliations:** 1 School of Psychology, Faculty of Medical Sciences, Newcastle University, 2.27 Ridley Building 1, Queen Victoria Road, Newcastle upon Tyne, NE1 7RU UK; 2 Department of Psychiatry, Autism Research Centre, University of Cambridge, Cambridge, UK

**Keywords:** Autism, Autistic traits, Anxiety, Depression, Anorexia nervosa, Bulimia nervosa, Eating disorders, Disordered eating, Sex differences, Gender differences

## Abstract

Previous studies have reported positive correlations between autistic traits and disordered eating, though it is unclear whether the association is moderated by sex/gender or whether it is independent of anxiety or depression. We present the findings of an online survey of 691 participants who completed the Autism Spectrum Quotient (AQ), Hospital Anxiety and Depression Scale (HADS), and Eating Attitudes Test-26 (EAT-26). Following a pre-registered analysis plan, we observed positive correlations between AQ and EAT-26 in males and females, with the association being significantly stronger in females. AQ also remained a significant predictor of EAT-26 when anxiety and depression were controlled for statistically. These findings may be relevant when considering therapeutic interventions in disordered eating populations that exhibit autistic traits.

## Introduction

Autism spectrum conditions are characterised by difficulties in social communication, restricted and repetitive behaviours, and sensory hyper- or hypo-sensitivities (American Psychiatric Association 2013). For a diagnosis to be made, symptoms must also have been present from early childhood, and must not be explainable by the presence of other conditions such as global developmental delay. Slightly more than 1% of the UK population is autistic (Baron-Cohen et al. 2009), approximately four males are diagnosed per every one female (Fombonne 2009), and co-occurring conditions such as anxiety and depression are common (Hollocks et al. 2019). In recent times, autism has typically been conceptualised as a dimensional condition representing one end of a continuum, and so traits associated with autism can be measured in non-clinical populations (e.g. with the Autism Spectrum Quotient [AQ]; Baron-Cohen et al. 2001). Evidence for the veracity of this conceptualisation comes from observations that autistic traits are normally distributed throughout the general population (Ruzich et al. 2015), that autistic individuals typically score considerably higher than neurotypical individuals on autistic traits measures (Baron-Cohen et al. 2014; Greenberg et al. 2018; Ruzich et al. 2015), and that the scores of first degree relatives of autistic individuals are intermediate to those of autistic and neurotypical people (e.g. Wheelwright et al. 2010). As with autism diagnoses, males typically report higher levels of autistic traits compared to females (Ruzich et al. 2015), and positive correlations are observed between autistic traits and symptoms of anxiety and depression (Kanne et al. 2009; Rosbrook and Whittingham 2010). The exact nature of the association between autistic traits and depressive/anxious symptomatology remains unclear, as, although autistic people are known to report higher rates of depression and anxiety compared to neurotypical people (e.g. Griffiths et al. 2019), people with depression (Domes et al. 2016) and anxiety (Hoekstra et al. 2008) also report higher levels of autistic traits than neurotypical controls. However, it appears that the elevated rates of anxiety and depression in autistic adults are partially mediated by an increased occurrence of negative life experiences (Griffiths et al. 2019).

Similarly to autism, eating disorders are frequently found to be comorbid with anxiety (Bulik et al. 1997; Griffiths et al. 2019; Hudson et al. 2007; Martín et al. 2019) and depressive disorders (Griffiths et al. 2019; Hudson et al. 2007; Martín et al. 2019). Anorexia nervosa (AN) and bulimia nervosa (BN), but not binge-eating disorder (BED), also exhibit marked sex/gender differences in their prevalence. Although the sex ratio for lifetime prevalence of AN and BN is typically reported as being 10 females per 1 male (Raevuori et al. 2014), the extent of this difference is partially attenuated in epidemiological studies (see Murray et al. 2017). In contrast to what is observed for autism, symptoms of eating disorders are less likely to be recognised in males, and are more likely to remain undiagnosed (Murray et al. 2017). Eating disorders are increasingly conceptualised trans-diagnostically, whereby a core psychopathology (over-evaluation of eating, weight, shape and their control, and dietary restriction and restraint) underpins disordered eating across the diagnostic spectrum (Fairburn et al. 2003; Linardon 2017). Moreover, as with autism and autistic traits, symptoms of disordered eating can be detected in non-clinical groups, i.e. symptoms are distributed dimensionally not categorically in the population (Keel et al. 2007). As such, many people exhibit disordered eating symptoms which do not reach the diagnostic threshold (Favaro et al. 2003; Reba-Harreleson et al. 2009). Reflecting this understanding of disordered eating, composite symptom measures such as the Eating Attitudes Test-26 (EAT-26; Garner et al. 1982) are typically used in research with non-clinical populations to capture a broad range of characteristic behaviours and cognitions. Extant research indicates that sex/gender differences in disordered eating symptoms mirror those observed for clinical eating disorders (e.g. Bartholdy et al. 2017; McCabe and Vincent 2003; Yu et al. 2018), and, in a similar manner, disordered eating symptoms correlate positively with symptoms of anxiety and depression (e.g. Abrams et al. 1993; Diehl et al. 1998; McCabe and Vincent 2003; Porter et al. 2018).

Due to a certain degree of symptom overlap, an association between autism spectrum conditions and eating disorders has been posited for several decades (Gillberg 1983, 1985), and there has been renewed interest in this idea in recent years (Odent 2010; Oldershaw et al. 2011; Treasure 2013). Due to similarities regarding cognitive inflexibility, impaired social functioning, and restrictive and repetitive behaviours, it has even been suggested that AN “might be considered a female variant of the autistic spectrum” (Odent 2010, p. 79), and recent evidence suggests that autistic women with eating disorders experience these conditions as being closely intertwined (Brede et al. 2020). A systematic review by Huke et al. (2013) reported a substantially increased prevalence (22.9%) of autism diagnoses within clinical AN and BN populations. Although a systematic review of more recent literature (Westwood and Tchanturia 2017) indicates that prevalence estimates may be lower in younger samples and research using parent-report measures, it is notable that a very similar proportion of women recruited from in-patient or day-patient specialist eating disorder services (14/60; 23.3%) scored above the clinical cut-off for autism on the ADOS-2 (Westwood et al. 2017). This suggests that even though the prevalence of diagnosed autism is elevated in eating disordered populations, many others who remain undiagnosed (Brede et al. 2020) could warrant a diagnostic assessment for autism. An autism diagnosis could potentially benefit such individuals by providing access to relevant services, increasing personal insight, and informing clinicians of their patients’ specific needs.

Several studies (e.g. Baron-Cohen et al. 2013; Hambrook et al. 2008; Tchanturia et al. 2013) have reported elevated levels of autistic traits in clinical eating disordered populations relative to healthy controls, with, a subsequent meta-analysis (Westwood et al. 2016) (n = 328 AN patients; n = 1890 healthy controls) revealing a large effect size (Cohen’s *d* = 1.065, 95% CI 0.83, 1.23) and no evidence of publication bias. The assessment of autistic traits within eating disordered populations may also be of further importance, as scores on the AQ-10 (a shorted version of the AQ that is used in clinical settings; Allison et al. 2012) in a population of AN patients were correlated with higher anxiety and depression, and with lower ability to maintain close relationships (Tchanturia et al. 2013). Although AQ-10 scores did not correlate significantly with the level of disordered eating symptoms in this sample, Fornaro et al. (2019) reported a significant (negative) correlation between AQ scores and BMI in a sample of n = 60 AN patients who were experiencing current depressive episodes. However, Solmi et al. (2020) demonstrated significant correlations between autistic traits and disordered eating even after BMI had been controlled for statistically.

There has been debate as to the exact nature of the association between autistic traits and disordered eating, as increased rigidity may be expected with decreased BMI due to the effects of starvation on the brain (Hiller and Pellicano 2013). However, a recent longitudinal study (Solmi et al. 2020) demonstrated that elevated autistic social traits measured at 7 years of age predicted disordered eating symptomatology at age 14, whereas disordered eating symptoms at age 14 did not predict autistic social traits measured at age 16 once baseline values and covariates had been controlled for. Furthermore there was evidence that the association between autistic traits at 7 years of age and disordered eating at 14 years was dose-dependent, with those exhibiting more frequent disordered eating behaviours having higher levels of autistic traits than those whose disordered eating behaviours occurred less frequently. Taken together, these findings suggest that elevated autistic traits in childhood are a risk factor for the later emergence of disordered eating pathology (Solmi et al. 2020).

It is important to consider that the majority of research participants in studies of eating disorders are female (Murray et al. 2016), and that results may therefore not be generalisable to males (Murray et al. 2017). Furthermore, the prevalence of eating disorders in males may be rising (Mitchison and Mond 2015; Murray et al. 2017). Murray et al. (2016, p. 414) suggest that the prevalence of eating disordered behaviour in men far outweighs the treated prevalence; these authors also suggest the reasons for this may be that males are particularly unlikely to seek treatment for eating disorders, because female-centric symptoms of such conditions in males are unlikely to be detected by health professionals (e.g. due to poor awareness, and/or because diagnostic instruments have typically been developed with stereotypical female presentations in mind), and because muscularity-oriented disordered eating is not currently considered a clinical eating disorder. Notably, Griffiths et al. (2015) reported that being male and having high levels of self-stigma of seeking psychological help were both predictive of the presence of undiagnosed eating disorders; furthermore, there was a significant interaction between these predictor variables, with the association between self-stigma and undiagnosed eating disorders being stronger in males than in females. Furthermore, males have been reported to have relatively poor ‘mental health literacy’ (Mond 2014), and the symptom profile for eating disorders can differ considerably between males and females (Murray et al. 2017). Notwithstanding those men who present with female-typical symptoms, such as significant drive for thinness and weight loss, there is increasing evidence to suggest that many other males present with a pursuit of muscularity (Mitchison and Mond 2015; Murray et al. 2016). For instance, Mangweth et al. (2001) showed that many male bodybuilders exhibit an obsessive pattern of eating and exercising similar to that of individuals with eating disorders but with the focus on gaining muscle rather than losing fat.

The above evidence suggests that more men experience eating disorders than is currently realised, and that this is to some degree contingent on ineffective recognition of symptoms more typically present in males, as well as the female-centricity of current diagnostic criteria (Mitchison and Mond 2015). However, the opposite pattern may be true of autism spectrum conditions. Biological variables such as differences in foetal sex hormone exposure (Auyeung et al. 2013; Baron-Cohen 2003; Baron-Cohen et al. 2015, 2019) may influence the sex/gender difference observed for diagnostic prevalence, and indeed sex/gender differences are known to be present for some core symptoms of autism. For example, autistic males have been reported to exhibit more restrictive and repetitive behaviours compared to autistic females (Wilson et al. 2016). However, stereotyping processes are also known to occur: autistic symptoms are less likely to be recognised in females than males (see Bargiela et al. 2016), and this effect appears to be particularly evident in autistic women with eating disorders (Brede et al. 2020).

A distinct female autism phenotype has been proposed, which may be frequently overlooked due to a bias in recognition for autistic features stereotypically associated with males. Experimental studies that manipulate sex/gender in vignettes of hypothetical children have shown that primary educators report being less likely to seek further support for females (as opposed to males) that exhibit symptoms typical of a female autistic presentation (Whitlock et al. 2020), and that laypeople are more likely to predict future atypicality in boys (as opposed to girls) who exhibit autistic features at an early age (Geelhand et al. 2019). Additionally, autistic females are thought to ‘camouflage’ their symptoms to a greater degree that autistic males (Lai et al. 2017), meaning that they may be less likely to come to clinical attention and so remain undiagnosed. Taken together, these observations suggest that although absolute sex differences appear to exist, the gaps between prevalence and detection may be magnified by gender stereotyping processes. For instance, if disordered eating symptoms are seen as a more socially accepted expression of autism-like traits in females (and indeed females may also be under greater pressure to conform socially), similar symptom profiles may result in different diagnoses in men and women.

Considering that autism may be underdiagnosed in females, that eating disorders may be underdiagnosed in males, and that clinical samples likely incur sampling bias, it is necessary to examine associations between autistic traits and disordered eating in non-clinical populations. Positive correlations have been reported between the AQ and the EAT-26 in a sample of 132 (61 males, 71 females) 11- to 14-year-old school children without recorded psychiatric diagnoses (Coombs et al. 2011), and in a sample of 80 (equal number of males and females) 18- to 25-year-old university students (Carton and Smith 2014). Mansour et al. (2016) then reported a similar correlation in a larger sample (n = 416; 82% female) of university students, as well as evidence to suggest the association was partially mediated by emotion dysregulation (but not by negative attitudes toward emotional expression). Although Raynal et al. (2016) did not observe a statistically significant difference in AQ-10 scores between French university students scoring above 20 on the EAT-26 (n = 101; 87.3% females, 12.7% males) and the rest of their sample (n = 378; 77.2% females, 21.8% males), the effect was in the expected direction (*d* = 0.16), and a recent systematic review (Christensen et al. 2019) concluded that a positive association does exist between these variables.

Given that there are widely reported sex/gender differences in the prevalence of autism and eating disorder diagnoses, it is noteworthy that only one study (Solmi et al. 2020) has so far examined associations between autistic traits and disordered eating in males and females separately. It also remains unclear whether associations between autistic traits and disordered eating are independent of anxiety and depression. To address these gaps in the literature, we present the findings of a pre-registered study (https://osf.io/mvx75/) that examined the potential moderating influences of sex/gender, anxiety, and depression, on the association between autistic traits (AQ) and disordered eating behaviour (EAT-26). Our main hypotheses were that AQ and EAT-26 scores would be positively correlated in both males and females, and that the association would be independent of concurrent symptoms of anxiety or depression. We assessed over-evaluation of weight and shape as a covariate in these analyses because the construct may help differentiate between obsessional/rigid approaching to eating that are characteristic of AN and BN (and possibly autism) from those characteristic of BED. Considering the apparently strong symptom overlap in women (Brede et al. 2020), we predicted that the association between autistic traits and disordered eating would be stronger in females than males.

## Method

### Sampling and Recruitment

We first conducted an a priori power analysis with G*Power 3 (Faul et al. 2009; Faul et al. 2007) to determine the required sample size. Using a previously reported effect size for the correlation between autistic traits and disordered eating (*r* = 0.26; Mansour et al. 2016), we determined that a sample of n = 113^1^ would be required to observe a statistically significant effect (two-tailed Pearson’s correlation) with 80% power and alpha set at *p* < 0.05. However, this was an underestimate considering that we intended to examine the association separately in males and female, and to control for several covariates. We therefore aimed to collect data from as many participants as possible during November and December 2018.

The survey was advertised on social media (e.g. Twitter), and also via the School of Psychology Research Participation Scheme, Newcastle University. As we intended to examine sex/gender differences in the association between autistic traits and disordered eating, and because males are often underrepresented in this type of research, efforts were made to oversample male participants. More specifically, we made sure to advertise the survey to relevant university departments (e.g. School of Engineering) and clubs and societies (e.g. Men’s Basketball). Participants from the general population were given the opportunity to enter a prize draw to win a £25.00 Amazon voucher, whereas Psychology students were offered course credits. Ethical approval for the study was granted by the Faculty of Medical Sciences Research Ethics Committee, Newcastle University (Approval Number: 8308/2018).

Eight hundred and fifty-one participants accessed the survey, 691 of whom completed at least one of the questionnaire measures (i.e. the AQ [at least one subscale], EAT-26 [at least one subscale], HADS [at least one subscale], Over-evaluation of Weight and Shape subscale from the EDE-Q [Fairburn and Beglin 1994, both items]) (missing values for these measures ranged from for 3.62% [Over-evaluation of Weight and Shape] to 5.64% [AQ total score]). Of these, n = 419 (60.6%) were female, n = 267 (38.6%) were male, n = 1 (0.1%) was transsexual, n = 1 (0.1%) preferred not to report their sex, and n = 3 (0.4%) responded to the question with ‘other’. With its emphasis on sex/gender differences, these latter n = 5 participants were not included in subsequent analysis. Of the 686 participants who had completed at least one of the outcome measures and reported that they were either male or female, 528 (77.0%) were students, 158 (23.0%) were not students, and ages ranged from 18 to 70 years (*M* = 23.63, *SD* = 10.14). Of those for whom it could be calculated (n = 665), BMI ranged from 11.99 to 60.35 (*M* = 23.42, *SD* = 4.46) (note that one impossible value [BMI = 0.00] was removed). The prevalence of relevant self-reported clinical conditions in this sample was as follows: autism (diagnosed, n = 9, 1.3%; suspected, n = 32, 4.7%), anxiety (diagnosed, n = 106, 15.5%; suspected, n = 171, 24.9%), depression (diagnosed: n = 108, 15.7%; suspected, n = 95, 13.8%), eating disorder (e.g. AN, BN, BED) (diagnosed, n = 21, 3.1%; suspected, n = 37, 5.4%). However, some participants responded with both ‘diagnosed’ and ‘suspected’ for the same condition(s), meaning that the overall prevalence of diagnosed and/or suspected conditions was as follows: autism (n = 40, 5.8%), anxiety (n = 260, 37.9%), depression (n = 188, 27.4%), eating disorder (n = 57, 8.3%). Note that the prevalence of these conditions could have been higher, as it was not possible to differentiate negative responses from missing values; however, the prevalence rates could also have been lower, as they are based on self-report and it was not possible to corroborate these data with those of confirmed clinical diagnosis.

### Design

The current study utilised a cross-sectional correlational design. The predictor variable was autistic traits, and the outcome was disordered eating symptoms.

### Materials/Apparatus

Participants were asked to report their age, sex (‘Female’, ‘Male’, ‘Intersex’, ‘Transsexual’, ‘Prefer not to say’, or ‘Other [please specify]’), age, occupation, height (in either centimeters or feet and inches), and weight (in either kilograms or stones and pounds). Students were required to select their level of study (‘University undergraduate’, ‘University postgraduate’ or ‘Other [please specify’]) and their subject area. Participants were also asked to specify whether they had been diagnosed with an autism spectrum condition, anxiety, depression, or an eating disorder. If they did not have an official diagnosis, participants could indicate whether they suspected they had any of these conditions.

The Autism Spectrum Quotient (AQ; Baron-Cohen et al. 2001) was used to quantify autistic traits. The measure is comprised of 50 items, approximately half of which are reverse-coded, and scores can range from 0 to 50. The scale has good test–retest reliability (Baron-Cohen et al. 2001) and can differentiate between autistic adults and neurotypical adults (Baron-Cohen et al. 2001; Woodbury-Smith et al. 2005). Internal consistency in the current study was high for the total score (Cronbach’s α = 0.840), though somewhat lower for the subscales (Social Skill, α = 0.730; Attention Switching, α = 0.651; Attention to Detail, α = 0.583; Communication, α = 0.652; Imagination, α = 0.519).

The Eating Attitudes Test-26 (EAT-26; Garner et al. 1982) was used to measure attitudes toward eating and food. The scale consists of 26 items on a six-point scale ranging from ‘Never’ (0) to ‘Always’ (6) (note that item 26 is reverse-scored), has reasonable test–retest reliability at 2–4 week follow-up (men *ICC* = 0.67; women *ICC* = 0.85; Forbush et al. 2019), and can differentiate between females with and without AN (Garner et al. 1982). Construct validity in women has been extensively demonstrated (e.g. Koslowsky et al. 1992) and the scale shows acceptable convergence with related measures in adolescent (McCreary and Sasse 2000) and adult (Tylka et al. 2005) males. The scale is comprised of three subscales: Dieting, Bulimia and Food Preoccupation, and Oral Control. Internal consistency in the current study was high for the total score (α = 0.895) and for the Dieting subscale (α = 0.899), though lower for the Bulimia and Food Preoccupation (α = 0.648), and Oral Control (α = 0.627) subscales.

In addition to the EAT-26, we used the two-item Over-evaluation of Weight and Shape subscale of the Eating Disorder Examination Questionnaire (EDE-Q) (Fairburn and Beglin 1994) to measure the extent to which weight and shape influenced individuals’ self-evaluation. Participants were initially asked “Over the past 4 weeks, has your weight influenced how you feel about (judge) yourself as a person?” and “Over the past 4 weeks, has your body shape influenced how you feel about (judge) yourself as a person?” These items were answered on a 7-point scale (1 = ‘Not at all’, 2 = ‘Very slightly’, 3 = ‘Slightly’, 4 = ‘Somewhat’, 5 = ‘Moderately’, 6 = ‘Strongly’, 7 = ‘Very strongly’). From these two values, a single mean score was derived. The EDE-Q is a widely-used questionnaire-based assessment for disordered eating and this subscale has been used previously as a stand-alone measure of Over-evaluation of Weight and Shape (e.g. Mond et al. 2007).

The Hospital Anxiety and Depression Scale (HADS; Zigmond and Snaith 1983) is a questionnaire used to measure current traits of depression and anxiety. Participants responded to 14 statements using a four-point scale, the wording for which differs between questions. For example, under the statement ‘I feel as if I am slowed down’, the response options are (1) ‘Not at all’, (2) ‘Sometimes, (3) ‘Very often’, and (4) ‘Nearly all the time’, whereas the response options for ‘I still enjoy the things I used to enjoy’ are (1) ‘Definitely as much’, (2) ‘Not quite so much’, (3) ‘Only a little’, and (4) ‘Hardly at all’. The scale is comprised of two 7-item subscales: Anxiety, and Depression. In the current sample, the internal consistency for the Anxiety subscale was high (α = 0.841), though that for the Depression subscale was low (α = 0.500).

### Procedure

Participants were presented with information about the study and were required to provide informed consent before proceeding further. The demographics questions were presented first, though the order of presentation for the other scales was randomised to reduce order effects. For the written version, participants undertook the same process except they were given a paper copy of the study materials and questionnaires. The consent form was edited so that students could sign each clause. Unfortunately, the scales were not randomised in this version of the study, meaning that all participants answering via pen and paper were presented with the scales in the same order (AQ, Over-evaluation of Weight and Shape, EAT-26, HADS). Participants were thanked and debriefed on completion, and signposting was provided towards online resources relating to mental health, autism, and eating disorders. Participants who completed the survey online (i.e. not Psychology students receiving course credits) were given the option to enter a prize draw for a £25.00 Amazon voucher.

### Data Analysis

The analysis plan was pre-registered on the Open Science Framework (https://osf.io/mvx75/), and the analyses presented here deviate from these initial plans only where specified. Body mass index was calculated as BMI = weight (kg) / [height (m)]^2^, and analyses were conducted using IBM SPSS version 26. As regression-based procedures are reasonably robust to non-normally distributed data when a large sample size is present (e.g. Williams et al. 2013), we use parametric statistics and untransformed values in all analyses. Effects are considered statistically significant at *p* < 0.05, and effect sizes are interpreted in accordance to the criteria specified by Cohen (1988): small (*d* = 0.20, *r* = 0.10, φ = 0.10), medium (*d* = 0.50, *r* = 0.30, φ = 0.30), or large (*d* = 0.80, *r* = 0.50, φ = 0.50).

## Results

### Sex/Gender Differences

Sex/gender differences were examined using independent samples *t* tests (Table 1). Males scored higher on the AQ and each of its subscales (differences for Social Skill and Communication subscales were not statistically significant), whereas females scored higher on the EAT-26 and each of its subscales (difference for Oral Control subscale was not statistically significant). Females also scored significantly higher than males on the HADS Anxiety subscale and on Over-evaluation of Weight and Shape, although there was no sex/gender difference for the HADS Depression subscale.

**Table 1:** Descriptive statistics and sex/gender differences for main study variables

	Females			Males			Difference			
	*N*	*M*	*SD*	*N*	*M*	*SD*	*t*	*df*	*p*	*d*
AQ total score	397	15.98	7.44	251	18.19	7.28	− 3.716	646	**< 0.001**	− **0.300**
AQ social skill	399	2.16	2.20	251	2.48	2.27	− 1.786	648	0.075	− 0.144
AQ attention switching	401	4.57	2.26	251	5.11	2.38	− 2.898	650	**0.004**	− **0.234**
AQ attention to detail	399	4.58	2.17	251	5.10	2.18	− 2.978	648	**0.003**	− **0.239**
AQ communication	401	2.38	2.02	251	2.65	2.18	− 1.597	650	0.111	− 0.130
AQ imagination	401	2.30	1.82	251	2.85	1.83	− 3.737	650	**< 0.001**	− **0.302**
EAT-26 total score	398	11.11	11.67	254	6.30	6.98	6.589	647.543^§^	**< 0.001**	**0.476**
EAT-26 dieting	402	7.34	7.88	255	3.70	5.28	7.088	653.070^§^	**< 0.001**	**0.521**
EAT-26 bulimia and food preoccupation	402	1.99	3.02	255	0.96	1.95	5.308	654.797^§^	**< 0.001**	**0.388**
EAT-26 oral control	402	1.72	2.75	254	1.63	2.08	0.517	632.692^§^	0.605	0.036
HADS anxiety	407	9.55	4.36	253	7.97	4.16	4.607	658	**< 0.001**	**0.369**
HADS depression	405	6.30	2.69	253	6.26	2.55	0.203	656	0.839	0.015
Over-evaluation of weight and shape total	403	4.17	1.85	259	3.05	1.75	7.794	660	**< 0.001**	**0.618**

^§^Indicates equal variances not assumed; effects displayed in bold are statistical significance (i.e. p < 0.05)

### Associations Between Study Variables

Partial correlations (controlling for age, sex/gender, BMI, and data collection method [online or pen/paper^2^]) were calculated to examine the associations among the main study variables. For ease of reporting, these analyses include only those participants for whom all of the measures included within this correlation matrix were present. Each correlation was in the positive direction, all but two (AQ Imagination and EAT-26 Oral Control; AQ Imagination and Over-evaluation of Weight and Shape) were statistically significant at *p* < 0.05 level, and 63 of 78 were significant at *p* < 0.001 (see Table 2).

**Table 2:** Intercorrelations between main study variables

	1	2	3	4	5	6	7	8	9	10	11	12
AQ total (1)												
AQ social skill (2)	0.766***											
AQ attention switching (3)	0.741***	0.455***										
AQ attention to detail (4)	0.556***	0.187***	0.278***									
AQ communication (5)	0.785***	0.639***	0.460***	0.257***								
AQ imagination (6)	0.593***	0.342***	0.302***	0.157***	0.355***							
EAT-26 total (7)	0.254***	0.135**	0.222***	0.223***	0.159***	0.127**						
EAT-26 dieting (8)	0.224***	0.103*	0.198***	0.205***	0.137**	0.122**	0.946***					
EAT-26 bulimia and food preoccupation (9)	0.210***	0.125**	0.177***	0.140**	0.156***	0.121**	0.766***	0.615***				
EAT-26 oral control (10)	0.177***	0.126**	0.155***	0.178***	0.093*	0.040	0.584***	0.373***	0.307***			
HADS anxiety (11)	0.410***	0.337***	0.413***	0.197***	0.315***	0.124**	0.290***	0.278***	0.230***	0.151***		
HADS depression (12)	0.429***	0.407***	0.322***	0.181***	0.338***	0.223***	0.259***	0.226***	0.226***	0.172***	0.499***	
Over-evaluation of weight and shape total (13)	0.199***	0.117**	0.186***	0.196***	0.131**	0.038	0.589***	0.608***	0.444***	0.209***	0.287***	0.209***

All analyses are partial (parametric) correlations (two tailed; df = 597) controlling for age, sex/gender, BMI, and data collection method (online or pen/paper)

*AQ* Autism Spectrum Quotient, *EAT*-*26* Eating Attitudes Test-26, *HADS* Hospital Anxiety and Depression Scale

***p < 0.001, **p < 0.010, *p < 0.050

### Moderating Effect of Sex/Gender on the Correlation Between Autistic Traits and Disordered Eating

**Fig. 1:**
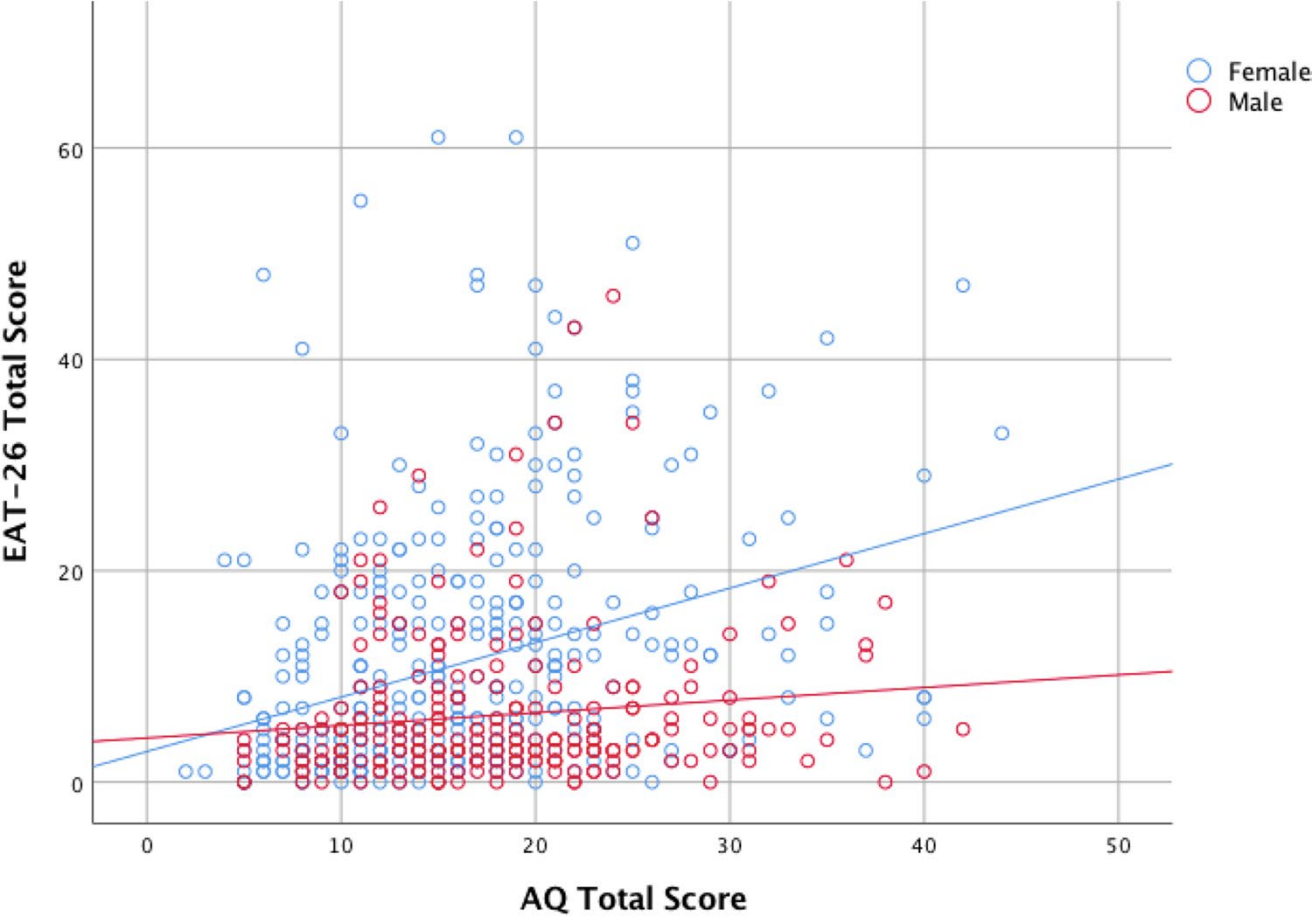
Scatterplot of the association between AQ total score and EAT-26 total score stratified by sex/gender. *AQ* Autism Spectrum Quotient, *EAT*-*26* Eating Attitudes Test-26

Having already established that AQ total score and EAT-26 total score were significantly positively correlated (*r*_partial_ = 0.254, *p* < 0.001; see Table 1), we next examined whether this relationship was present in males and females separately. Partial correlation analysis (controlling for age, BMI, and data-collection method) revealed a significant positive association in females, *r*_partial_(363) = 0.322, *p* < 0.001, and a similar trend towards significance in males, *r*_partial_(239) = 0.111, *p* = 0.087 (Fig. 1). A Fisher’s *r*-to-*z* transformation confirmed our hypothesis that the correlation between AQ total score and EAT-26 total score would be significantly stronger in females than in males, *z* = 2.66, *p* = 0.008 (two-tailed).

### Autistic Traits, Anxiety, and Depression as Independent Predictors of Disordered Eating

We conducted multivariate regression analyses (using the enter method) to determine whether the association between autistic traits and disordered eating was independent of anxiety and depression. At the suggestion of a reviewer, we modified our analyses by entering the AQ*sex interaction term into the models to further clarify the existence of a moderating effect of sex/gender, and by using a hierarchical approach to determine how much additional variance in EAT-26 score could be predicted by the HADS subscales after taking into account the influence of AQ and the other covariates. We entered the predictors/covariates in the following order: Step 1: AQ total score; Step 2: sex, AQ*sex, age, BMI, data collection method, and Over-evaluation of Weight and Shape; Step 3 HADS Anxiety and AQ*Anxiety; step 4: HADS Depression and AQ*Depression (see Table 3).

**Table 3:** Hierarchical linear regression models of the association between autistic traits (AQ total score) and disordered eating (EAT-26 total score)

	Step 1				Step 2				Step 3				Step 4			
	Unstandardized coefficients		Standardized coefficients		Unstandardized coefficients		Standardized coefficients		Unstandardized coefficients		Standardized coefficients		Unstandardized coefficients		Standardized coefficients	
	B	95% CI	Beta	p	B	95% CI	Beta	p	B	95% CI	Beta	p	B	95% CI	Beta	p
(Constant)	4.106	2.119, 6.092		< 0.001	− 5.126	− 11.581, 1.328		0.119	− 6.701	− 13.224, − 0.179		0.044	− 6.858	− 13.377, − 0.339		0.039
AQ total	0.300	0.192, 0.408	0.217	< 0.001	0.654	0.392, 0.915	0.473	< 0.001	0.643	0.371, 0.914	0.465	< 0.001	0.609	0.336, 0.882	0.441	< 0.001
Sex/gender					4.080	0.752, 7.407	0.195	0.016	4.514	1.175, 7.853	0.215	0.008	4.385	1.046, 7.724	0.209	0.010
AQ*sex/gender					− 0.327	− 0.503, − 0.152	− 0.471	< 0.001	− 0.340	− 0.517, − 0.162	− 0.489	< 0.001	− 0.336	− 0.513, − 0.159	− 0.484	< 0.001
Age					− 0.030	− 0.094, 0.035	− 0.030	0.367	− 0.020	− 0.085, 0.045	− 0.020	0.546	− 0.022	− 0.087, 0.043	− 0.023	0.502
BMI					− 0.180	− 0.329, − 0.031	− 0.079	0.018	− 0.185	− 0.333, − 0.036	− 0.081	0.015	− 0.198	− 0.347, − 0.048	− 0.087	0.010
Over-evaluation					3.154	2.788, 3.520	0.580	< 0.001	3.055	2.680, 3.429	0.562	< 0.001	3.033	2.659, 3.407	0.558	< 0.001
Data collection					− 1.065	− 2.780, 0.649	− 0.041	0.223	− 1.022	− 2.729, 0.685	− 0.039	0.240	− 1.037	− 2.741, 0.668	− 0.040	0.233
Anxiety									0.205	0.033, 0.378	0.085	0.020	0.135	− 0.050, 0.320	0.056	0.152
AQ*anxiety									− 0.014	− 0.033, 0.004	− 0.049	0.133	− 0.018	− 0.042, 0.006	− 0.061	0.150
Depression													0.303	− 0.003, 0.609	0.075	0.052
AQ*depression													0.005	− 0.035, 0.045	0.010	0.814
Model fit	*F* (1, 601) = 29.744, *p* < 0.001				*F* (7, 595) = 61.272, *p* < 0.001				*F* (9, 593) = 48.936, *p* < 0.001				*F* (11, 591) = 40.559, *p* < 0.001			
*R*^2^	0.047				0.419				0.426				0.430			
*R*^2^ change					0.372				0.007				0.004			
*F* change					*F* (6, 595) = 63.436, *p* < 0.001				*F* (2, 593) = 3.767, *p* = 0.024				*F* (2, 591) = 2.069, *p* = 0.127			

*AQ* Autism Spectrum Quotient, *HADS* Hospital Anxiety and Depression Scale, *BMI* Body Mass Index

Step 1 determined that AQ score was a significant (positive) predictor of EAT-26 scores, though it only accounted for a small amount of variance (*R*^2^ = 0.047). Entering covariates at Step 2 resulted in a significant improvement, with the model then able to account for a much larger portion of variance (*R*^2^ = 0.419, *R*^2^ change = 0.372, *p* < 0.001). Entering HADS Anxiety and AQ*Anxiety at Step 3 resulted in a very small but statistically significant increase in predictive value (*R*^2^ = 0.426, *R*^2^ change = 0.007, *p* = 0.024). Adding Depression and AQ*Depression to the model at Step 4 resulted in a very small increase in variance explained that was not statistically significant (*R*^2^ = 0.430, *R*^2^ change = 0.004, *p* = 0.127). This final model (Step 4) determined that there were six independent predictors of high EAT-26 scores: AQ (high scores), sex (female), BMI (low values), Over-evaluation of Weight and Shape (high scores), AQ*sex/gender interaction term (stronger association between AQ and EAT-26 in females than males), and HADS Depression (although this last predictor was only marginally significant, *p* = 0.052). A closer inspection of the models presented in Table 3 reveals that at Step 3, HADS Anxiety was a significant (positive) predictor of EAT-26 scores, though drops out of the model at Step 4; at this point it is essentially replaced by HADS Depression, which appears to explain a very similar proportion of variance in the outcome variable.

### Association Between Diagnosed/Suspected Autism and Diagnosed/Suspected Eating Disorders

As an additional (i.e. not pre-registered) analysis, we examined whether the presence of diagnosed and/or suspected autism was associated with the presence of diagnosed and/or suspected eating disorders. Although the effect was in the expected direction (i.e. with the conditions co-occurring more often than expected by chance), a Chi square test showed that it was not statistically significant, χ^2^ (1, 686) = 2.496, *p* = 0.114, φ = 0.060.

## Discussion

The main aim of the current study was to investigate associations between autistic traits and disordered eating in a general population sample of males and females. In accordance with previous findings, we observed statistically significant sex/gender differences for the AQ, EAT-26, Over-evaluation of Weight and Shape, and HADS Anxiety. Notably, however, we did not detect a significant sex/gender difference for HADS Depression, though this finding should be considered in light of the low internal consistency (α = 0.500) observed for this measure in the current study. As predicted, statistically significant (positive) correlations were detected between almost all combinations of these variables. However, the findings of greatest interest were that we observed a significant positive correlation between AQ total score and EAT-26 total score in females (*r*_parial_ = 0.322, *p* < 0.001) and a similar trend towards significance in males (*r*_partial_ = 0.111, *p* = 0.087), that the correlation was significantly stronger in females than in males, and that it remained statistically significant in the combined (i.e. male + female) sample after controlling for concurrent anxiety and depression.

Previous research (Carton and Smith 2014; Coombs et al. 2011; Mansour et al. 2016) has reported statistically significant correlations between autistic traits (AQ total score) and disordered eating (EAT-26 total score) in non-clinical samples. However, other than for one recent study (Solmi et al. 2020) there has been little consideration for a possible moderating effect of sex/gender. The study by Solmi et al. (2020) reported that the pattern of association was similar in boys and girls, though notably lacked the statistical power required to examine associations by frequency of disordered eating behaviour in males. The current study is therefore the first to show that this association is significantly stronger in females than in males. These findings are important considering that autism spectrum conditions and eating disorders both exhibit marked sex/gender differences in their diagnosis rates, that males are typically underrepresented in eating disorder research (Murray et al. 2016), and that females are underrepresented in autism research (Lai et al. 2015). In particular, these findings may be relevant when considering the propensity for symptoms to go undetected, unrecognised, and undiagnosed in autism (females > males) and eating disorders (males > females).

Although it may be that autistic traits and disordered eating really are more strongly correlated in females than in males, sex/gender differences in the phenotypic expression of eating disorders (and autism) may be relevant in explaining this observation. For instance, it has been suggested that the typical eating disorder symptom profile in males may reflect a drive toward muscularity rather than the drive toward thinness more typically observed in females (Murray et al. 2016), and that processes involved in the development and maintenance of eating disorders in autistic women may be quite different from those typically observed in non-autistic women (Brede et al. 2020). It is also relevant to note that disordered eating screening measures such as the EAT-26 often include female-centric questions that may not resonate strongly with males (Darcy et al. 2012). An obvious extension to the current research would therefore be to examine whether autistic traits are correlated with symptoms of muscularity-oriented disordered eating, and, if so, whether this correlation is conversely stronger in males than in females.

Previous studies (Carton and Smith 2014; Coombs et al. 2011; Mansour et al. 2016) have reported positive correlations between various combinations of AQ subscales (Social Skill, Attention Switching, Attention to Detail, Communication, Imagination) and EAT-26 subscales (Dieting, Bulimia and Food Preoccupation, Oral Control). This has led to some speculation about which domains may be most integral in explaining the overall association between autistic traits and disordered eating. However, our study casts doubt on there being strong domain specificity, as statistically significant positive correlations were observed between all combinations of these subscales except for AQ Imagination and EAT-26 Oral Control (*r*_partial_ = 0.040, *p* = 0.332. For the inter-subscale correlations that were statistically significant, the strength was from negligible (lowest *r*_*parial*_ = 0.093, *p* = 0.022 for AQ Communication and EAT-26 Oral Control) to small (highest *r*_*partial*_ = 0.205, *p* < 0.001 for AQ Attention to Detail and EAT-26 Dieting), and none was as strong as the correlation between AQ total score and EAT-26 total score (*r*_*partial*_ = 0.254, *p* < 0.001). We also observed a positive correlation between AQ total score and Over-evaluation of Weight and Shape (*r*_*partial*_ = 0.199, *p* < 0.001); as with the correlations for the EAT-26, this effect appeared not to be domain specific, with statistically significant positive correlations of small magnitude being observed between Over-evaluation of Weight and Shape and each of the AQ subscales other than Imagination.

It has been suggested that social communication difficulties present from an early age may lead to anxiety and depression, which may then lead to the development of disordered eating (Solmi et al. 2020), and also that dietary restriction might be used by some autistic women as a maladaptive coping strategy to reduce anxiety (Brede et al. 2020). However, our study found that AQ total score remained a statistically significant (positive) predictor of disordered eating behaviour after controlling for anxiety and depression. Considering that autistic traits, disordered eating, anxiety, and depression have all be reported to correlate positively with each other, it is important to have determined that AQ scores are independently associated with disordered eating, and that this effect is not simply a statistical artefact. Essentially, whereas previous studies have established the existence of these intercorrelations, we show here that the association between autistic traits and disordered eating remains statistically significant once anxious/depressive symptomatology is held constant. These findings are therefore similar in nature to those of Mealey et al. (2014), who established that anxiety and depression do not account for the overlap between AQ scores and schizotypal traits, and suggest that the correlation between autistic traits and disordered eating cannot be explained by there being a simple lack of specificity in the AQ (see Hoekstra et al. 2008). Awareness of the current findings may encourage practitioners to consider autistic traits in patients displaying disordered eating symptoms. As a result, treatment may be individualised to patients with elevated levels of both autistic traits and disordered eating, potentially bringing clinical benefits such as reduced recovery periods and prevention of symptom escalation. This is of particular relevance when considering the observation of Tchanturia et al. (2016) that AN patients with low levels of autistic traits responded positively to group Cognitive Remediation Therapy (CRT), whereas those with high levels of autistic traits did not. Such findings led Westwood and Tchanturia (2017, p. 1) to conclude that “co-morbid AN and ASD may require more intensive treatment or specifically tailored interventions”, and suggest that a better understanding of the association between these conditions is required if more effective clinical interventions are to be developed.

### Strengths and Limitations

There are several strengths to our study. First, our large sample provided sufficient statistical power to reliably detect small to medium sized effects. Second, we incorporated a relatively well-balanced number of males (n = 267, 38.9%) and females (n = 419, 61.1%), which enabled us to examine sex/gender as a moderator. Third, we controlled for other relevant covariates, including anxiety and depression; this is an important consideration because, although anxiety and depression are known to be associated with both autistic traits and disordered eating, most previous studies have not included them in their statistical models. Fourth, we implemented Open Science practices by pre-registering our hypotheses and analysis plan before collecting data.

The current study also incurred some limitations. Firstly, it relied on self-report measures of autistic traits, anxiety, depression, and disordered eating. Although this facilitated collection of a relatively large dataset, it does mean that we relied on participants being able to provide accurate accounts of their current mental health status. This is an important consideration because, although some people are insightful and self-aware, others may underestimate or overestimate the severity of their current symptoms. Future research may provide more accurate insights into the nature of the association between autistic traits and disordered eating by interviewing participants’ relatives and health professionals to obtain reliability estimates. Additionally, it was noted that the majority of missing data for the current study came from participants who completed the survey via pen and paper rather than online. Although this is consistent with the observation that electronic surveys tend to result in fewer missing data than do print surveys (Boyer et al. 2002), this effect may have been exacerbated by the fact that the online study prompted participants to provide answers when relevant, whereas this was not possible in the pen and paper version. It may therefore be beneficial to use electronic as opposed to pen and paper surveys in this area of research.

Another limitation to our study is that inclusion criteria required participants to be at least 18 years of age. Although this is standard practice in much psychological research, it may be a specific problem in regard to eating disorders. This is because adolescent females represent a group at particularly high risk of experiencing eating disorders (e.g. Smink et al. 2012), yet many of those within this demographic would have been ineligible to take part in the current study. Excluding this younger age group is therefore likely to have resulted in an underestimation of the prevalence of eating disorders found in the general population. The generalisability of our findings may also have been reduced somewhat by the relatively high prevalence of Psychology students within our sample, and also by our deliberate oversampling of males from specific university courses and societies.

## Conclusion

Overall, the findings of this study provide further evidence of there being a positive correlation between autistic traits and disordered eating. The study also builds on previous research by demonstrating that the association is present in both males and females, that the correlation is stronger in females, and that AQ scores can predict EAT-26 scores independently of concurrent symptoms of anxiety and depression. The study therefore provides a foundation upon which clinical research may gain a better understanding of the nature of the association between autism and eating disorders, and may be useful for the formulation of therapeutic interventions. Additionally, increased awareness of the association between autistic traits and disordered eating means that clinicians working with autistic and/or eating disordered populations may consider screening patients during early assessments. Although necessarily speculative at this point, this process could potentially provide clinicians with information relevant for determining effective intervention strategies at a relatively early stage of treatment.

## Data Availability

The datasets used and/or analyzed in the current study are available from the study’s Open Science Framwork page (https://osf.io/mvx75/).

## Footnotes

1 Note that this was incorrectly reported as n = 111 in the pre-registration.

2 Note that in our pre-registered analysis plan we did not specify that we would enter data collection method as a covariate. However, we chose to do this after considering that those participants who completed the questionnaires via pen and paper differed from the other participants in four main ways: (1) they completed the questionnaires in a group setting, (2) they completed the questionnaires via pen and paper rather than online, (3) they were all first and second year undergraduate Psychology students, (4) they completed the survey in exchange for course credits and so were ineligible to enter the prize draw. Due to these observations, we included data collection method (dichotomised as online vs. pen and paper) as a covariate in each of the multivariate analyses presented in the current study.
